# Chimeric aptamers in cancer cell-targeted drug delivery

**DOI:** 10.3109/10409238.2011.614592

**Published:** 2011-09-28

**Authors:** Jagat R Kanwar, Kislay Roy, Rupinder K Kanwar

**Affiliations:** Nanomedicine Laboratory of Immunology and Molecular Biomedical Research (LIMBR), Centre for Biotechnology and Interdisciplinary Biosciences (BioDeakin), Institute for Technology and Research Innovation (ITRI), Geelong Technology Precinct (GTP), Deakin University, Geelong, Victoria, Australia

**Keywords:** Aptamer, SELEX, chimera, targeted drug delivery, si RNA, locked nucleic acid, nanoparticles

## Abstract

Aptamers are single-stranded structured oligonucleotides (DNA or RNA) that can bind to a wide range of targets ("apatopes") with high affinity and specificity. These nucleic acid ligands, generated from pools of random-sequence by an *in vitro* selection process referred to as systematic evolution of ligands by exponential enrichment (SELEX), have now been identified as excellent tools for chemical biology, therapeutic delivery, diagnosis, research, and monitoring therapy in real-time imaging. Today, aptamers represent an interesting class of modern Pharmaceuticals which with their low immunogenic potential mimic extend many of the properties of monoclonal antibodies in diagnostics, research, and therapeutics. More recently, chimeric aptamer approach employing many different possible types of chimerization strategies has generated more stable and efficient chimeric aptamers with aptamer-aptamer, aptamer-nonaptamer biomacromolecules (siRNAs, proteins) and aptamer-nanoparticle chimeras. These chimeric aptamers when conjugated with various biomacromolecules like locked nucleic acid (LNA) to potentiate their stability, biodistribution, and targeting efficiency, have facilitated the accurate targeting in preclinical trials. We developed LNA-aptamer (anti-nucleolin and EpCAM) complexes which were loaded in iron-saturated bovine lactofeerin (Fe-blf)-coated dopamine modified surface of superparamagnetic iron oxide (Fe_3_O_4_) nanoparticles (SPIONs). This complex was used to deliver the specific aptamers in tumor cells in a co-culture model of normal and cancer cells. This review focuses on the chimeric aptamers, currently in development that are likely to find future practical applications in concert with other therapeutic molecules and modalities.

## Introduction

On the therapeutic front, in the race for developing targeted delivery vehicles, nucleic acid-based aptamers are now regularly in competition with small molecules and antibodies since the inception of aptamer technology more than a decade ago. Aptamers (Latin *aptus* meaning “to fit") are the functional nucleic acid ligands generated by a molecular selection process called Systematic Evolution of Ligands by Exponential Enrichment (SELEX) and are also one of only a few classes of molecules that similar to antibodies can be crafted to bind to multiple different targets (Keefe, 2008; Kanwar *et al*., 2010c). Aptamers are 20-80 base pair long single-stranded deoxy ribonucleic acid (DNA) or ribonucleic acid (RNA) oligonucleotides which are folded into unique three-dimensional conformations due to various intramolecular interactions (Kanwar *et al*, 2010c).

The idea of usingoligonucleotides as therapeutic agents is not a new one; as in 1990 RNA decoys, which mimic the transactivating responsive (TAR) RNA of human immunodeficiency virus (HIV), were shown to prevent HIV replication by sequestering the Tat protein (Sullenger *et al*, 1990; Bivalkar-Mehla *et al*, 2010). However, among the various nucleic acid-based strategies employed for drug discovery research and therapeutics, aptamers have emerged as the most promising agents for therapy and diagnosis, since the origin of aptamer technology two decades ago (Ellington, 1990; Tuerk & Gold, 1990). Aptamer-mediated drug delivery enhances the therapeutic efficacy due to their high specificity to the target and thus their affinity reduces the off-target effects or other unwanted side effects, commonly observed with widely used cytotoxic drug therapeutics. In regard to comparison with traditional antibodies, aptamers also known as “chemical antibodies,” have number of advantages such as (i) smaller in size and less complex with low immunogenic potential, (ii) easier to synthesize and modify *in vitro*, (iii) higher affinity and specificity, (iv) structural flexibility enabling aptamer to bind to hidden epitopes which cannot be targeted by antibodies (Majumder, 2009), and (v) stronger stability and can be stored easily until put to use. As the use of aptamers has been extended from basic biology of cellular processes and gene regulation to therapeutic and diagnostic applications, many patented aptamers are currently being tested in clinical trials and reviewed recently (Majumder, 2009). The resulting outcomes will provide important validations of their efficacies and cost saving. The approval of an anti-vascular endothelial growth factor (VEGF) aptamer (Eyetech/Pfizer's Macugen) six years ago, by the Food and Drug Administration (FDA) for treatment of age-related human macular degeneration has already proved a milestone for the applications of aptamer technology. In this review, we focus on the importance of aptamer and their chimerics in the field of molecular medicine. The review focuses on the various selection procedures along with the established modifications for the aptamers and their chimerics. Various forms of aptamer-chimerics have been discussed with their therapeutic benefits and strategies for targeted delivery. Some of the aptamers in clinical trials are also listed.

## Strategies for aptamer selection

For screening of aptamers, the target and the characteristic of aptamers obtained must be known; a random pool of oligonucleotides both DNA and RNA have to be made for screening; the immobilized targets are used for screening of aptamers from the RNA or DNA pool (Wang, 2009). The specificity of the aptamer and its ability to distinguish between the closely related species depends on the type of selection procedure for its isolation. SELEX technique developed by Tuerk, Ellington, and Szostak was the first aptamer selection procedure (Tuerk & Gold, 1990; Ellington & Szostak, 1992). It involves selection of nucleic acid ligands which interacts with specific target in a repeated binding, selection, and amplification of aptamers from initial library until the desired characteristics have been isolated. First, an oligonucleotide library is created which has sequences that can be amplified by polymerase chain reaction (PCR). The library can be used directly for DNA-based aptamer selection, whereas for selection of an RNA aptamer, the DNA library has to be converted into a RNA library; the known target molecule is then incubated in the oligonucleotide pool along with the target accompanied with heating and cooling to promote formation of stable structures. The oligonucleotides bound to the target are isolated and are put through repeated rounds of selection for obtaining the sequences with the best specificity. These sequences are then cloned in plasmids, amplified, sequenced and known as aptamers (Tuerk & Gold, 1990).

In 1992, Ellington and Szostak (1992) discovered another screening method for aptamers with a 10-fold higher affinity called as the negative SELEX, where the nucleotide pool was loaded on a matrix with analog of the target, and the unbound sequences are then used to incubate with the target for normal binding. Thus, aptamers selected by negative SELEX had higher affinity to the target and could discriminate between the closely related targets. Negative SELEX has been used for selection of small molecules (Bassett *et al*., 2004) and proteins (Haller & Sarnow, 1997). Jenison and co-workers (1994) established another screening methodology called as counter SELEX, for screening of small molecules’ aptamers where the cycles were similar to negative SELEX; however, here the matrices were exchanged with the analogs of the target molecules. Wang and co-workers (2003) discovered subtractive SELEX which removes single-stranded DNA or RNA sequences that can bind to nonspecific part of a complex target with various binding sites in order to obtain highly specific aptamers in the end. This process has extensively been used to screen aptamers for various cancers.

There are many diagnostic markers and therapeutic targets expressed on the cell surface. The aptamers selected by SELEX may show low or no affinity toward them due to shielding of the aptamer's binding domain. To overcome this, several groups, as discussed in the review by Xianbin and co-workers (2011), have reported the isolation of aptamers from living cells as targets such as cancer cells (Shangguan *et al*., 2006) by the process called as cell-SELEX. In cell-SELEX, instead of using a purified protein target, whole living cells are used as the targets. Live African and American trypanosomes aptamers (Homann & Göringer, 1999; Ulrich *et al*., 2002) are some of the examples of cell-SELEX. The only drawback in this process is that it has to be repeated more than 25 times (Jenison *et al*., 1994; Sooter *et al*., 2001). Capillary electrophoresis (CE-SELEX)-based selection procedure developed by Bowser and Krylov groups is another revolution in field of aptamer selection (Berezovski, 2004; Mendonsa & Bowser, 2004). Here the oligonucleotide library is mixed with the target molecule and introduced into a free solution CE system. The unbound oligonucleotides migrate at a different rate than the oligonucleotides which are bound to the target. The collected bound sequences are then amplified by PCR and high affinity aptamers can be obtained only after two rounds of selection (Mendonsa & Bowser, 2004). An advancement to the CE-SELEX is the micromagnetic selection (M-SELEX), which uses magnetic beads as solid support matrix for linking the target (Bruno & Kiel, 2002). More recently, the number of beads can be manipulated to isolate high affinity aptamers. The DNA aptamers against the light chain of botulinum neurotoxin type A has been isolated in a single round of selection by this method (Qian *et al*., 2009).

The other forms of SELEX are covalent SELEX (Jensen *et al*., 1995), which is used to isolate aptamers that bind covalently to the target moiety, Toggle SELEX (White *et al*., 2001) that generates aptamers with the ability of cross reactivity in different species, bead-based SELEX (Yang *et al*., 2002a), Tailored SELEX (Vater *et al*., 2003), which was able to regulate the size of aptamers by removing the fixed nucleotide sequences from the oligonucleotide library, and on chip SELEX (Collett *et al*., 2005). More innovatively, Miyachi *et al*. used atomic force microscopy based SELEX (AFM-SELEX) which has the capability to detect the force of affinity or adhesion between two molecules, to isolate anti-thrombin aptamers within just three rounds of selection (Miyachi *et al*., 2010).

With time the aptamer selection procedures have evolved due to the developing technology. Better techniques have been devised to select aptamers with high affinity and maximum specificity to the desired target. However, the use of aptamers in diagnosis of biological fluids has been limited. Thus, post-SELEX changes and chemical modifications have made their way into aptamer technology due to a continuous need of stability and efficient activity.

## Established aptamer modifications to overcome aptamer instability

The use of modified nucleotides has been the most commonly approached method to overcome aptamer instability. Precise site-specific modifications facilitate engineering of aptamers for delivery into target cells with enhanced specificity. The need for modification is justified by functional optimization, truncation, or multimerization of aptamers which has enhanced the binding efficacy and stability. Various linkage designs, modification strategies, and conjugation approaches are prevalent in aptamer technology. The various modifications of nucleotides, mainly the chemical ones, are compatible with the enzymatic steps of *in vitro* selection procedure, introduced either at phosphate/ribose backbone or at the nucleobases (Keefe & Cload, 2008).

## Chemical modifications in aptamers

Replacement of DNA phosphate backbone by phosphorothionate enhanced stability against nucleases and the cell viability of aptamers (Eckstein & Gish, 1989). However, most prominent modification of aptamers is derivatization of 2'-ribose, as this position conferred stability of most RNA aptamers (Yang *et al*., 2002b). Several other different approaches have been reviewed for improving aptamer stability in biological fluids due to the sensitivity of both DNA and RNA aptamers to nucleases which limits their therapeutic and diagnostic potential (Kusser, 2000). Most of these approaches rely on the incorporation of nucleotides carrying modifications of either sugar residues (2'-F, 2'-NH_2_ (amino), 2'-O-methyl, 2'-O-methoxyethyl and 2'-O-dimethyl allyl) (Green *et al*., 1995; Ruckman *et al*., 1998) of the phosphate (phosphorothioate and methyl phosphonate) (Eckstein & Gish, 1989) or of the nitrogenous base [propenyl, 5-(N amino-alkyl) carbomoyluracil, methyl, trifluoromethyl, phenyl and 2-thiopyrimidine] (Lin *et al*., 1996).

**Figure 1:**
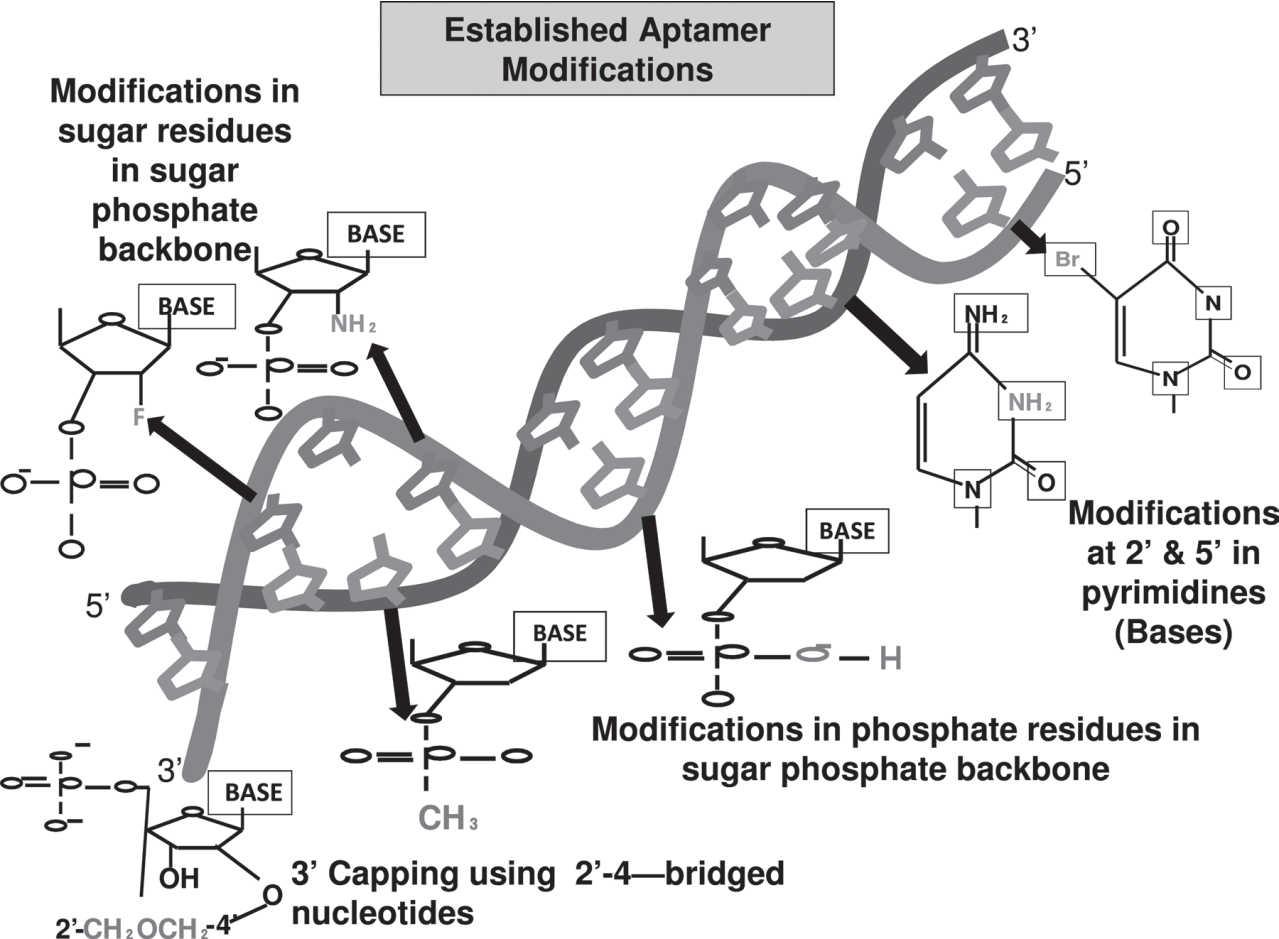
Figure shows the various modifications made in an aptamer to increase its stability and functionality. The common modifications made in the ribose residue of the sugar-phosphate backbone are incorporations of 2'-fluorine (F) and 2'-amino group (NH2), in the phosphate group of the sugar-phosphate backbone are incorporation of phosphorothioate and methyl phosphonate. The modification made in the nucleotide base includes incorporation of 5'- bromide (Br) and amino group (NH2) (Cheung et al., 2010). The 3'-capping of the aptamer sequence is also another form of modification, which helps in increasing the stability of aptamer and its prevention from nuclease degradation (Kasahara et al., 2010). (See colour version of this figure online at www.informahealthcare.com/bmg)

Pyrimidines are modified at fifth position to enhance the stability of the aptamer with iodide (I), bromide (Br), chloride (Cl), amino (NH_3_), azide (N_3_) (Kirschenheuter, 1997) and 2’ position with amino (NH_2_), fluoro (F), and methoxy (OCH_3_) (Barry *et al*., 1996). These modifications also provide nuclease resistance. The 2’ sugar modifications, fifth position pyrimidine modification, substitution of 4-thiouridine, substitution of 5-bromo or 5-iodouracil, backbone modification, methylations and unusual base pairing combinations such as isobases, isocytidine, and isoguanodine (Sampson, 2003), and 3’ capping (Kasahara Y, 2010) are other forms of modifications which provide thermal stability and nuclease resistance to aptamers (Figure 1). The SELEX protocol also offers possibility of further diversity during selection and enrichment where modifying the aptamers is much easier when compared to post-SELEX methods (Sampson, 2003).

Aptamers with photolabile protecting groups offer a possibility for the spatiotemporal regulation of aptamer activity with control over activity of the target molecule. Using this modification the aptamer can be activated or deactivated by specific light intensity (Mayer *et al*., 2005; Heckel *et al*., 2006). Hence aptamers can be modified to fluoresce at their fifth position by chemical or enzymatic synthesis (Sengle, 2000). The chemical modifications are induced to increase the half life and stability of the aptamers. The modified aptamers can be obtained by spiegelmer technology, which is also known as mirror image SELEX. In this method, aptamers of chiral target from naturally occurring dextrorotatory oligonucleotide pool are screened and from them the complementary levorotatory oligonucleotide is synthesized called as spiegelmers. These spiegelmers have equivalent capability as the initial aptamer to bind with high affinity to the target (Wlotzka *et al*., 2002) and the chemical modification is induced later as the levorotatory molecule with chemical modifications cannot be recognized by SELEX enzymes; this molecule can recognize the target with more specificity and has better stability (Eulberg & Klussmann, 2003).

## Chimeric aptamer: an insight

There is an increasing enthusiasm for generating new and more potent cell internalizing, highly specific aptamers with greater stability and nuclease resistance. Recombination is a powerful evolutionary force and it is a known fact that the protein segments are routinely recombined to engineer various chimeric proteins with novel functions. Aptamer diversity is required for the development of more established and better characterized aptamers; thus the need for modification and chimerization arises. Chimerization started with the idea of creating antibodies for any potential target (Riechmann *et al*., 1988; Russell *et al*., 1992). The constant need for higher specificity, stability, and effectiveness motivated better synthesis procedures over time. Similarly to antibodies and proteins’ chimerization, aptamer chimerization has been carried out for characterizing and diversifying them over a long period of time (Goldstein *et al*., 1995; Song *et al*., 2005).

Chimerization of aptamers refers to its combination with another aptamer, biomacromolecule(s) and/or compounds where the functional capability of the chimera is a combined factor of aptamer as well as the other biomacromolecules and/or compounds used in combination (Burke & Willis, 1998).

Chimeric aptamer approach aims to combine two aptamers or an aptamer with another non-aptamer moiety (biomacromolecules, drug, or dyes), where one molecule engages with the target and the other has a functional effect on the target molecule. When two aptamers or an apatmer with another biomacromolecule are added together, either through natural recombination or chemical engineering, it is difficult to know beforehand whether the joining may diminish the activity of one or both of the recombining partners; hence, chimerization was considered as a challenging procedure (Burke & Gold, 1997a; Burke & Willis, 1998). However, with the progress of research in this field as discussed here, it is very clear that chimeric aptamers are not only highly stable and efficient in activity but are also able to deliver drug loads.

## Selection of the aptamer chimeras

The selection of chimeric aptamer over the parent type aptamer needs efficient comparison between them in terms of specificity of binding and the binding affinity. Within a chimeric SELEX, two or more different libraries are used for the production of chimeric aptamers with more than one wanted feature or which are able to function in different ways. Each of the parent libraries will be selected first for a distinct feature. The selected biomolecules are then fused in such a way that they acquire several properties (Burke & Willis, 1998). Chimeric SELEX method simulates random recombination among functional aptamers derived from nucleotide population with 70 to 80 positions of random sequences as a mean to generate aptamers with novel functions. Another method to obtain multifunctional aptamers is multiple SELEX; two aptamers with specificity toward coenzyme-A, chloramplenicol or adenosine could be fused. This selection strategy was applied to obtain a suitable chimera capable of binding both the targets. This will have several applications such as catalysis, therapeutics, structure studies, etc. (Burke *et al*., 1997b; Burke & Willis, 1998) In another work, two nucleic acid pools of 40 and 60 units were used to screen aptamers for cibacron blue and cholic acid. After five to six rounds of selection, the aptamers were fused and the ones with the ability to bind both cibacron blue and cholic acid were obtained. It was observed that the chimeric aptamer could bind either to cibacron blue and could be eluted by cholic acid or could bind to cholic acid and be eluted by cibacron blue; these results highlighted the importance of allosteric interactions in mechanism of chimerism (Wu & Curran, 1999).

Zinc fingers have diverse functions in a cell, mainly dealing with interactions with DNA, RNA, or proteins and play a major role in the regulation of gene expression (Laity *et al*., 2001). Work done in this field compared interaction of several different zinc finger proteins with RNA1 (a high affinity aptamer for zinc finger) where, sitedirected mutagenesis in RNA1 was used to identify key and structural elements in RNA1 involved in the formation of high affinity interactions with a broad range of zinc fingers (Lu *et al*., 2003). The modified forms, RNA1, RNA21, and RNA 22, were compared to characterize RNA molecules with high affinity for a set of zinc fingers (Weiss *et al*., 2010). Such studies help in identifying the most specific aptamer for a target which has several applications such as coordinated release of protein on a specific time scale or release in a specific cellular location. A similar work describes isolation of 2'-fluoro (2'-F)-substituted RNA aptamers that bind to streptavidin with high affinity. After selection of aptamer SA19 (specific for streptavidin), it was mutated by DPTP [6-(2-deoxy-β-D-erythropentofuranosyl)-3, 4-dihydro-8H-pyrimido-(4, 5C) (1, 2) oxamine-7-one-5'-triphosphate], a nucleotide analog. The 2'-F-pyrimidine containing RNA transcripts from various mutagenesis cycles were analyzed by surface Plasmon resonance (SPR) to verify the abolition of binding to streptavidin. Metal transporting ATPases CopA and CopT sequences were inserted downstream of SA19 aptamer to form chimeric SA19-CopA or SA19-CopT. Their properties were then compared with SA19 unmodified aptamer for characterization of binding affinity and specificity (Alaoui *et al*., 2002). In the study done by McNamara and co-workers, even though the first generation prostrate specific membrane antigen (PSMA) aptamer/ polo-like kinase 1(Plk1)-siRNA (A10-Plk1) chimera inhibited tumor growth, it lacked the ability to be systemically administered (McNamara *et al*., 2006). Thus, in continuation of their work, a second generation of PSMA-Plk1 chimeras was designed to facilitate chemical synthesis, to enhance silencing activity, specificity, and to enable modifications to optimize *in vivo* kinetics. The changes made to the first generation chimeras to facilitate chemical synthesis were to reduce the aptamer size from 71 nucleotides to 39 nucleotides and a 2 ‘-F was added to the longer strand and the shorter strand was unmodified. To increase the silencing activity, several chimeras were engineered, ones with an overhang at 3’ end of siRNA duplex, ones with a wobble base at 5’ end of guiding strand of siRNA, ones where the passenger and the guide strand were swapped, and ones with a stem loop. Then, the binding of optimized chimeras to PSMA expressing cells was tested; all chimeras had retained the binding ability confirming that modifications made to first generation did not alter binding or specificity. To determine the enhancement of silencing activity of the chimeras for gene-specific silencing, a quantitative real time polymerase chain reaction (qRT PCR) was used and it was confirmed that siRNA portion of chimera enhanced Plk1 silencing, most active were swap and stem-loop chimeras. Finally, the effect of chimeras on growth and survival of prostate cancer cells was tested and it was found that second generation chimeras inhibited cell growth and proliferation at a lower concentration than the first generation chimeras (Justin *et al*., 2009). Even though they are engineered well and have more capability, the chimeric aptamers are as prone to degradation as an unmodified aptamer. However, there are some molecules such as locked nucleic acids (LNA's) (Lebars *et al*., 2007), which when incorporated into the chimeras provide a higher grade of stability and specificity and thus help in the selection of the chimeras by giving them an additional advantage over the parental types.

## LNA-based modifications

**Figure 2:**
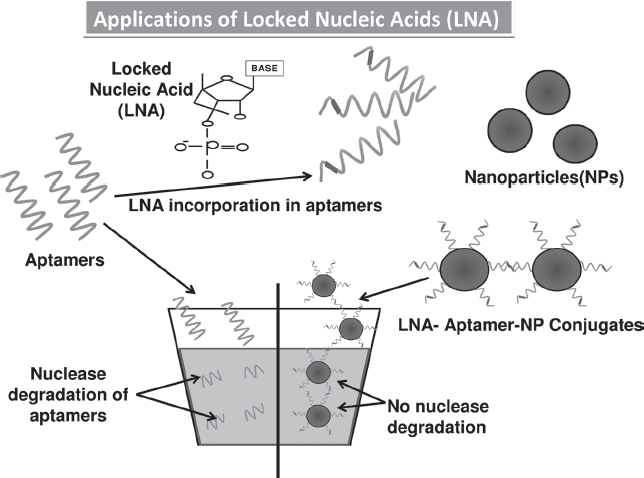
The structure of the locked nucleic acid molecule is shown in Figure 3. The reason for its stability is due to the presence of an additional bond between the hydrogen on the 4th carbon and the oxygen atom on the 2nd carbon, which also gives the molecule a locked structure and makes it more resistant to thermal degradation. The incorporation of the LNA molecules into the aptamer is also shown which stabilizes the structure, and finally the LNA-incorporated aptamers are loaded onto a nanoparticle for target delivery (DI Primo *et al*., 2007; Lebars *et al*., 2007; Seferos *et al*., 2007; Barciszewski *et al*., 2009). (See colour version of this figure online at www.informahealthcare.com/bmg)

Both DNA and RNA oligonucleotides are highly susceptible to degradation by nucleases. Thus, the potential applications and the usage of aptamers in drug delivery are limited by the susceptibility of aptamers to degradation by intracellular nucleases. To further improve the stability against nucleases and to overcome this limitation, the use of non-natural bases, such as LNA, were used (Orum & Wengel, 2001; Petersen & Wengel, 2003; Hernandez *et al*., 2009;). The LNA comprises of a new class of bicyclic high affinity analogs (2'-O, 4'-C-methylene-β-D-ribofuranosyl nucleotide) in which furanose ring of ribose sugar is chemically locked in an RNA mimicking conformation by introduction of 2'-O,4'-C-methylene bridge. These impart high degree of thermal stability when hybridized with their DNA and RNA target molecule (Figure 2). A specific feature of LNA is that they induce the neighboring DNA to adopt an A-type conformation in a DNA-RNA duplex. Since LNA hybrids are not compatible with standard enzymes that are used in SELEX, only post-SELEX is followed for their selection (Barciszewski *et al*., 2009). The introduction of LNA into aptamers renders the molecule highly resistant toward nuclease degradation. Significant stabilization of aptamers has been achieved by introduction of LNA at various positions of oligonucleotides. There are mainly three types of LNA: antigenic LNA, antisense LNA, and LNA aptamers. Immense work has been done with LNA hybrid aptamers, such as in a 39-mer oligonucleotide TTA1 (RNA aptamer); LNA was introduced which exhibited significant stem stabilization and improved plasma stability of the aptamer while maintaining high binding affinity to the target (Barciszewski *et al*., 2009). Another study showed that LNA was used for modification of R06 aptamer to generate stable hybrid with improved binding affinity to form kissing complexes with the target with higher nuclease resistance (Lebars *et al*., 2007). Recently invented LNA nucleotides have been shown to be effective both in and without combination of taxol for inhibiting expression of survivin thereby leading to tumor growth inhibition when compared to LNA nucleotides alone (Kanwar *et al*., 2010a). Survivin is a member of inhibitor of apoptosis (IAP) family of proteins that has a dual role in cell division (mitosis) and apoptosis (Kanwar *et al*., 2001; Altieri, 2003; Baratchi *et al*., 2010; Kanwar RK *et al*., 2010; Kanwar, 2010a). It is known to be over-expressed in most of the tumors and has become recently an attractive target for novel anticancer therapies (Kanwar *et al*., 2001; Altieri, 2003; Baratchi *et al*., 2010; Kanwar RK *et al*., 2010; Kanwar *et al*., 2010a). A set of researchers prepared locked nucleic acid nanoparticle conjugate (LNP's) by adding thiol-terminated LNA oligonucleotides to a solution containing gold nanoparticles (AuNPs) in phosphate buffer and also prepared DNA-modified nanoparticle analogs for comparative study by using antisense DNA and LNA, and they found out that LNP's were more stable and induced a better relative decrease in survivin expression (Seferos *et al*., 2007). In another work, chimeras were synthesized by surface plasmon resonance (SPR) from 64 LNA/2'-o-methyl sequence to all possible combination in a kissing RNA-aptamer loop complementary to epsilon-nucleotide loop of TAR element of HIV-1. Following which a chimera of higher specificity than the parent RNA aptamer was obtained and one of the chimeras also showed inhibition of TAR-dependant luciferase expression in a cell assay (DI Primo *et al*., 2007). The LNA incorporations have successfully changed the scenario of aptamer instability and their effectiveness has been well established with time.

**Figure 3:**
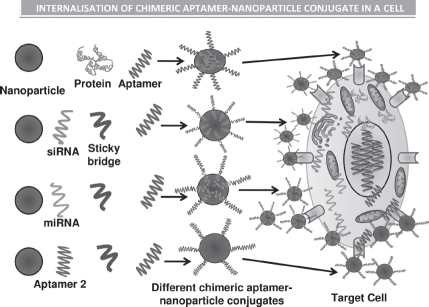
Illustrations of the various types of chimeric forms of aptamers established, such as the aptamer-antibody (Ohk *et al*., 2010), aptamer-protein (Zhou & Rossi, 2010), aptamer-siRNA (Kawata *et al*., 2010) (Pastor *et al*., 2010), aptamer-miRNA (Lunse, 2010), and the aptamer-aptamer (Held *et al*., 2006) chimeras. The use of sticky bridges is also depicted which helps in binding of the aptamer moiety to the siRNA and the miRNAs due to presence of complementary regions on the sticky loop for both siRNA/miRNA and the aptamer. The aptamer conjugates are then shown to conjugate with the nanoparticles binding to the target cells by the interaction of aptamer-receptor interaction, and finally the nanoparticles are internalized inside the target cells and results in release of siRNA, miRNA, protein, or the aptamer molecule, respectively (Lundberg *et al*., 2007; Farokhzad *et al*., 2004; Khaled *et al*., 2005; Alonso *et al*., 1994; Molpeceres *et al*., 1999). (See colour version of this figure online at www.informahealthcare.com/bmg)

In spite of being able to incorporate several beneficial properties into the chimeras, their delivery is yet another necessary stage which plays a key role in their action.

## Various types of aptamer chimeras and their functional applications in therapeutics

There are many possible types of chimerization such as aptamer-siRNA chimeras, aptamer-enzyme chimeras, and aptamer-aptamer chimeras (Table 1 and Figure 3). Aptamer technology has evolved in a multidirectional fashion finding importance and playing essential roles in fields of biosensing, imaging, drug delivery, and modern molecular medicine for therapy for various diseases and disorders.

**Table 1:** Chimeric aptamers and their action.

S. No.	Chimera type	Specific to	In *vitro/in vivo*	Action	Trial state	Reference
1.	Aptamer-antibody (Apt-A8-P66 Ab)	Internalin A of L. *monocytogenes*	*L. monocytogenes*	Detection of *L*. *monocytogenes* in food samples	Pre-clinical	Ohk *et al* (2010)
2.	Aptamer-aptamer (DNA Aptamer-RNA malachite green aptamer)	Adenosine	Cell free assay	Detection of adenosine	Pre-clinical	XU and LU (2010)
3.	Aptamer-enzyme (antitransferring aptamer-α-L-iduronidase)	Transferrin	*In vitro-mouse* fibroblast Ltk cells	Restores activity in cells lacking the enzyme	Pre-clinical	Chi-Hong *et al*. (2008)
4.	Aptamer-siRNA (A10 aptamer-siRNA)	PSMA	*In vitro-LNCaP* cells	Inhibits expression of PLK1 and BC12	Pre-clinical	Zhou and Rossi (2010)
5.	Aptamer-siRNA (PLK1aptamer-siRNA)	PLK1	*In vitro-*Human MM cells- H2452, H2052, H28, and 211H. NDHF cells	Inhibits expression of PLK1	Pre-clinical	Kawata *et al*. (2010)
6.	Aptamer-siRNA (PSMA apt-smgl/siRNA)	PSMA	*In vitro-CT26,B16 In vivo*-athymic nude mice	Inhibiting nonsense mediated mRNA decay	Pre-clinical	Pastor *et al*. (2010)
7.	Aptamer-siRNA (PSMA apt-siRNA)	PSMA	*In vitro*-PC-3, LNCaP, HeLa. *In* w'yo-athymic nude mice	Inhibits expression of PLK1	Pre-clinical	McNamara *et al*. (2006)
8.	Aptamer-siRNA	PSMA	*In vitro-HeLa*, N12-E, human cervical cancer	Boron neutron cancer therapy	Pre-clinical	Shaw *et al*. (2008)
9.	Aptamer-siRNA (PSMA apt-shRNA-Dox-PEG-PEI)	PSMA	*In vitro-PC-3*, LNCaP	Inhibits expression of Bcl-xl	Pre-clinical	Kim *et al*. (2010)
10.	Aptamer-siRNA (anti-TAR apt(R06)-siRNA)	TAR	Cell free assay	Inhibits TAR expression	Pre-clinical	Lebars *et al*. (2007)
11.	Aptamer-siRNA (anti- gpl20 apt-siRNA)	Gpl20(HIV)	*In vito*-HEK293, CEM, PBM cells donors.	Inhibits HIv-1 P24 production and gpl20 expression	Pre-clinical	Zhou *et al*. (2008)
12.	Aptamer-miRNA (Aptamer-pri-miRNA)	Pri-miRNA	*In vitro*-HeLa	Modulating pri-miRNA processing	Pre-clinical	Katoh and Katoh (2008)
13.	Aptamer-NRTI	HIV1-RT	*In vitro*-lymphoid cells lines	Inhibition of DNA polymerization by HIV1-RT	Pre-clinical	Held *et al*. (2006)
14.	Aptamer-thrombin	Mercury (2+) and Lead (2+)	Cell free assay	Detection of Hg(2+), Pb(2+)	Pre-clinical	Smirnov and Shaffer (2000)
15.	Aptamer-aptamer	ATP	Cell free assay	Detection of ATP	Pre-clinical	Chen *et al*. (2008)

PSMA, prostate specific membrane antigen; LNCaP, androgen sensitive human prostate carcinoma; PLK1, polo like kinasel; smgl, gene coding for serine/ threonine-protein kinase SMG1; HeLa, human cervical cancer cells; HEK293, human embryonic kidney 293 cells; CEM, human lymphoblastic cells; Human MM cell, human multiple myeloma cells; NDHF cells, normal human dermal fibroblast cells; PBM cells, peripheral blood mononuclear cells.

### Aptamer-biomolecule conjugate

A recent work done on aptamer-antibody chimera provides an effective methodology for sensitive and specific identification of a pathogenic bacteria *Listeria monocytogenes* in food. Aptamer A-8, specific for internalin A, an invasion protein of *L. monocytogenes* was used in fiber optic sensor together with biotinylated P66 antibody in a sandwich format for the detection of *L. monocytogenes* from food samples (Ohk *et al*., 2010). Many bacterial strains have become drug resistant with the overexposure to antibiotics worldwide. Bacterial infection is a major health concern and demands more advanced treatments, such as use of nanotechnology or nanomedicine (Mathews *et al*., 2010). Chimeric aptamers can play potential role in antibacterial diagnostics and therapeutics due to obvious benefits over traditional antibacterial medicines. An aptamer-aptamer chimera has been developed recently, containing adenosine DNA aptamer and malachite green (MG) RNA aptamer joined by bridging strands that has complementary sequences to both aptamers. In the presence of adenosine, aptamer strand binds to adenosine leaving fewer numbers of complementary base pairs between aptamer and bridge strand, which is less stable, and results in release of bridge strand and observance of fluorescence of malachite green (XU & LU, 2010). The fluorescence can be detected by eye and no sophisticated instrument is required for this. Such chimeras can be used very efficiently in real time detection and quantification of small analytes. Work done by another group shows an example of aptamer-enzyme chimeras, the aptamer against transferrin receptor was isolated and coupled to a dephos-phorylated α-L-iduronidase enzyme. It was shown that the aptamer-enzyme chimera was successfully taken up by receptor-mediated endocytosis and restored activity in cultured fibroblasts lacking this enzyme (Chi-Hong *et al*., 2008).

### Aptamer-biomacromolecule conjugate

The most established and best-characterized chimeras are the aptamer-siRNA chimeras. This interesting and recent innovative approach aims to use the aptamers in siRNA therapeutics. Though work has been done with these chimeras (Eli, 2010; Zhou *et al*., 2011), yet most prevalent one targeted for therapy is against PSMA, a cell-surface receptor over-expressed in prostate cancer cells and tumor vascular endothelium (McNamara *et al*., 2006). Some of the work done on PSMA is summarized here. An aptamer-siRNA chimera was developed where the aptamer portion of the chimeras mediated binding to PSMA, whereas the siRNA portion targeted the expression of tumor survival genes polo-like kinase 1 (Plk1)17 and Bcl-2. The chimera effectively delivered the associated siRNA specifically to LNCaP prostate cancer expressing PSMA cells and triggered apoptosis that resulted in cell death both in culture and in a prostrate tumor xenograft model (McNamara *et al*., 2006). In another study 2'-F modified anti-PSMA aptamer (A-10) was covalently linked to sense strand of 21-mer siRNA hybridized to 21-mer antisense strand, the resulting chimeric was shown to selectively internalize into cells expressing PSMA and inhibit expression of targeted tumor survival genes (Plk1 and Bcl2) aptamer. A portion of PSMA-A10PLK1 chimera was truncated from 71 to 39 nucleotides, while still maintaining high binding affinity; structural changes were made to siRNA enabling efficient incorporation (Zhou & Rossi, 2010; Zhou *et al*., 2011).

In a similar work, a DNA-chimeric siRNA was generated against Plk1, which was more stable in human serum than nonchimeric siRNA and the chimeric Plk1-siRNA inhibited malignant mesothelioma (MM) cell proliferation through the induction of apoptosis *in vitro*. This DNA-modified siRNA chimera was constructed by substituting six ribonucleotides at the 5’ end of the guide strand with the cognate de-oxyribonuclotides; thus the siRNA could be protected from RNAse or nuclease degradation by partial substitution of ribonucleotides with de-oxyribonucleotides (Kawata *et al*., 2010). Another recent work describes an alternative approach in which expression of new and potent antigens is induced in tumor cells by inhibiting nonsense-mediated messenger RNA decay (NMD). In this study, PSMA aptamer-smgl/siRNA chimera was made to stimulate protective antitumor immunity and it was suggested that the dose treatment of aptamer-siRNA chimeras can be further optimized to inhibit tumor (Pastor *et al*., 2010). To down regulate gene expression through tryptophanase (TNA) interference in combination with aptamertherapy a 21 -nucleotide double stranded (ds) siRNA and fluoro-modified aptamers were used. Substituting a borane phosphate for part of natural phosphodiester bond in RNA allows the use of siRNA to persist in cells for longer than unmodified siRNA and the boron neutron capture therapy (BNCT) to specifically kill prostate cancer cells (Shaw *et al*., 2008). Work done with aptamer-siRNA on other targets included use of anti-TAR aptamer (R06) as regulator of TAR-dependant gene expression to benefit affinity and resistance to RNAses (Duconge & Toulme, 1999). The binding between the aptamer and target occurs in form of kissing interactions as R06 has a hairpin RNA loop complementary to TAR and guanosine-adenosine (G-A) combination, stacking interactions and hydrogen bonds at stem/loop-loop junction aids in stability of complex (Duconge & Toulme, 1999; Lebars *et al*., 2007). Modification of R06 by N3’ to P5’ phosphoramidate, 2'-omethyl, hexitol nucleic acid, improved nuclease resistance; however, there was no increase in binding affinity (Lebars *et al*., 2007). A novel dual inhibitory functional anti-gp 120 aptamer-siRNA chimera, in which both aptamer and siRNA have potent anti-HIV activities has also been developed (Zhou *et al*., 2008). In this approach, anti-gp 120 aptamers (A-1 and B-68) were used which inhibited HIV-1 P24 production and provided more potent inhibition of HIV infectivity. The aptamer and siRNA were linked via sticky sequences consisting of 16 nucleotides at aptamer 3’ end, which were complementary to 16 bases on one of the two-siRNA strands (Zhou *et al*., 2008; Zhou *et al*., 2011). The sticky bridge base approach offered a major advantage in both the chemical synthesis and the opportunity to mix and match the aptamer with different siRNAs in a non-covalent fashion.

Micro RNAs (miRNAs) are short sequences of nucleotides occurring naturally in cells that are important in regulating gene function and expression of pathways by controlling the amount of miRNA that binds to a gene and is possible to upregulate or downregulate the genes (Vella & Slack, 2005). The miRNAs can be targeted to the stem cell signaling components or epithelial to mesenchymal transition (EMT) regulators and aptamers can be utilized for directing the application of miRNA as a potent therapeutic (Katoh & Katoh, 2008). Various miRNAs have tumor suppressing activity and may play a vital role in other disease prevention or development; hence, they are important in diagnosis and therapy of human diseases (Silahtaroglu A, 2010; Kanwar, 2010b). Isolation and characterization of RNA aptamers that specifically binds to primary transcript miRNA to the apical loop domain of the pri-miRNA has been done. The results showed that aptamers could be applied as agents for modulating pri-miRNA processing and also as tools for understanding the mechanism (Lunse, 2010).

### Aptamer-drug conjugates

A work done with aptamer-siRNA chimera dealt with the development of a chimeric molecule using PSMA aptamer with small hairpin RNA (shRNA) against anti-apoptotic factor BCL-XL and drug doxorubicin (dox). Their conjugation into a polyplex of polyethyleneimine-polyethyleneglycol (PEI-PEG) was done for target-specific delivery, synergistic, and selective cancer cell death by aptamer mediated co-delivery of doxorubicin and shRNA was achieved by activation of an intrinsic apoptotic pathway in prostate cancer cells, both *in vitro* and *in vivo* (Kim *et al*., 2010). Nucleotide analog reverse transcriptase inhibitors (NRTI), for example, Azidothymidine triphosphate (AZTTP) and 2', 3'-Dideoxy cytidine-5'-triphosphate (ddCTP), are chain terminators that prevent completion of viral genome replication following their incorporation in reverse transcriptase (RT) during DNA polymerization (Enomoto L, 2011). A recent work tested aptamers in combination with NRTI, which displayed significant synergy for inhibition of DNA polymerization by HIV1 -RT, where the RNA and DNA aptamers were generated by SELEX, specific for HIV-1 RT (Held *et al*., 2006).

### Aptamer-dye conjugates for nanodiagnostics

In presence of mercury, Hg(2+) and lead, Pb(2+) a thrombin-binding aptamer containing six thymine and nine guanine units changes into a hairpin like structure (Smirnov & Shaffer, 2000), thus thymine-containing thrombin-binding aptamers can be used to target mercury, Hg(2+) (Miyake *et al*., 2006). According to a recent chimeric approach, the domains of two aptamers can be linked, one that engages a fluorophore for signaling and other that binds a nonfluorescent target, into one sequence in such a way that the binding of the non-fluorescent target will strengthen or reduce the affinity of the chimeric aptamer for fluorophore. The signal is produced as a result of the aptamer-fluorophore association or separation that is accompanied by a change in fluorescence intensity. Similarly, multiple targets can be simultaneously reported by using two or more structure substituting aptamers labeled with different fluorophores, for example, the adenosine tri-phosphate (ATP) binding aptamer, ATP1.1, can form a signaling duplex with FDNA1 (carrying fluorescein) and QDNA2, while the guanosine tri-phosphate (GTP) binding aptamer, GTP 1.2, can form a signaling duplex with FDNA2 (carrying cy3) and QDNA2. The resulting mixture can generate a realtime signal for both ATP and GTP when both reporters are mixed into a single solution (Nutiu & Li, 2005).

To facilitate the diagnosis, identification, and study of mechanism of various diseases and pathways, highly efficient aptamer-based detection systems have also been validated. To detect the presence of cocaine in samples, the functionalized quantum dots (QDs) consisting of two subunits of aptamers were used, where one was linked to QD and the other was specific to cocaine and formed the cocaine-aptamer-aptamer-QD complex in presence of cocaine, where cocaine induced a conformational change in the complex leading to change in QD behavior (Freeman, 2009; Zhang & Johnson, 2009). Detection of ATP has been achieved by adsorption and covalent coupling of ATP-binding DNA aptamers onto cellulose, where the properties of cellulose enhance the DNA aptamer activity (Su *et al*., 2007). Another study introduced a new fluorescent method for detection of ATP using the following three different oligonucleotides in a complex; (i) A 3'-biotin modified DNA specific to streptavidin bound to streptavidin coated on QDs; (ii) 3'-cy5 labeled DNA; and (iii) an aptamer specific to ATP. In the absence of ATP, all three oligonucleotides formed a stable complex and thus normal fluorescence was observed, whereas, in the presence of ATP, the 3'cy5 labeled DNA got dissociated from the complex and the fluorescence intensity changed (Chen *et al*., 2008). Angiogenin is a potent angiogenic protein which has a role in tumor angiogenesis and RNA transcription (Xua *et al*., 2002). Cellular internalization of angiogenin was investigated by a highly specific fluorophore-labeled aptamer for angiogenin by real-time protein imaging in living cells using confocal laser scanning microscopy (Li, 2008). Other examples include detection of C-reactive protein (CRP) which is highly expressed in inflammation; it also serves as a biomarker (Tsimikas *et al*., 2006). The detection was based on a sandwich format where a biotinylated RNA aptamer specific to CRP was immobilized on streptavidin-coated magnetic beads; the modified beads were incubated with CRP solution and recoupled with same biotinylated aptamer. After the binding, the extent of affinity was evaluated by addition of enzymatic substrate, which was transformed into an electroactive product and the detection of the enzymatic reaction was done by differential pulse voltametry (DPV) (Centi *et al*., 2010). Super paramagnetic iron oxides (SPION) were used as nanoparticles; A10 RNA aptamer against PSMA was tagged with doxorubicin (dox) and covalently bound to the nanoparticles, which induced magnetization inside a magnetic field (Wang *et al*., 2008).

For imaging applications, the aptamers can be bound to imaging agents such as fluorophores, QDs, magnetic resonance imaging (MRI) agents, magnetic nanoparticles, etc. The method of visualization mainly depends on the type of nanoparticle used or the modifications made in the aptamer and aptamer chimerics which aid in their detection. In a recent work, the AS1411 aptamer specific to nucleolin protein was conjugated to cobalt-ferrite nanoparticles surrounded by fluorescent rhodamine within a silica shell and was incorporated into cancer cells for imaging. The imaging was based on magnetic fluorescence nanoparticles inside cancer cells by MRI (Hwang *et al*., 2010). Theophylline is a bronchodilator used in case of asthma, bronchitis, and emphysema; since its overdose can lead to toxic effects, it is important to detect its levels in body. The AuNP were conjugated to theophylline-specific aptamer. In the presence of theophylline, the aptamer dissociates from AuNP leading to an aggregation of AuNP and an increase in AuNP plasmonic peak. The observed intensity enables the detection of theophylline (Chavez *et al*., 2010). In another study, theophylline was studied and detected by ferrocene (Fc) redox-labeled RNA aptamer. The aptamer was able to quantify the theophylline in serum and thus proved to be an efficient biosensor (Ferapontova & Gothelf, 2009). Redox active probes are used in combination with aptamers, and by measuring the changes in their redox states, the electrochemical detection is carried out, such as in case of thrombin-bound aptamer (Smirnov & Shaffer, 2000). Lysozyme detection has been made possible by using an aptamer-functionalized silica nanoparticles where once the aptamer interacts with the protein, a fluorescence signal is emitted by anionic poly(fluorene-alt-vinylene) (PFVSO_3_) via electrostatic interaction. This detection can be done by naked eye and is a remarkable achievement in recent trend (Wang *et al*., 2010a) (Table 2). In another work, atomic force microscopy-based imaging of nucleosomal arrays was done by acetylation of histones at 16th lysine residue and recognized by a DNA aptamer specific for histones (Lin *et al*., 2009).

**Table 2:** Aptamer based detection systems.

S. No.	Target	Detection system	Importance	Reference
1.	Cocaine	Cocaine-aptamer-aptamer-QD	Detection of cocaine in various samples	Freeman (2009); Zhang and Johnson (2009)
2.	ATP	Cellulose-coated ATP-binding aptamer	Detection of ATP and study of various pathways involving ATP	Su *et al*. (2007)
3.	Angiogenin	Fluorophore-labeled aptamer	Study of tumor angiogenesis	Li (2008)
4.	C Reactive Protein (CRP)	Fluorophore-labeled aptamer	Detection of inflammation	Centi *et al*. (2010)
5.	Prostate specific membrane antigen (PSMA)	A10 RNA aptamer-DOX-SPION	Detection of PSMA in prostate cancer	Wang *et al*. (2008)
6.	Histones	DNA aptamer and atomic force microscopy	Structure and properties of histones	Lin *et al*. (2009)
7.	Nucleolus	AS 1411 aptamer-cob alt ferrite nanoparticles in silica shell	Cancer cell imaging and various purposes	Hwang *et al*. (2010)
8.	Theophylline	AuNP with theophylline specific aptamer	Detection of theophylline and study of asthma	Chavez *et al*. (2010)
9.	Theophylline	Ferrocene (Fc) redox labeled RNA aptamer	Detection of theophylline and study of asthma	Ferapontova and Gothelf (2009)
10.	Lysozyme	Aptamer functionalized silica nanoparticles with anionic conjugate polymer	Detection of lysozyme is various samples	Wang *et al*. (2010a)

Hence a lot of work has been done on various targets with different types of aptamer chimeras; however, the strategy and the parameters considered for their selection remains quite the same. The most successful and commonly used aptamer chimeras are the aptamer-siRNA chimeras, which have been used extensively to target various diseases.

## Mechanisms and strategies of delivery of chimeras

It is known that aptamers cannot enter the cell membrane directly by simple diffusion due to their hydrophobic nature; hence, various studies have been conducted to study the mechanism of entry of aptamer into a cell.

### Carrier peptides

Due to the poor permeability and selectivity of the cell membrane, there is a need of a strategy that can transport aptamers or drugs into large number of cells. The studies indicate that the mechanism of endocytosis best explains the intracellular trafficking of aptamers. Endocytosis and related pathways like classic clathrin-coated pit pathway, caveolar pathway, clathrin-independent noncaveolar pathway, macropinocytosis, and phagocytosis are the mechanisms that explain the entry of aptamers in cells (Kirkham & Parton, 2005; Perret *et al*., 2005). The complex flow of endomembrane traffic is regulated by Rab family of GTPases and by tethering complexes (Stenmark & Olkonnen, 2001), while vesicular fusion phenomenon is controlled by SNARE proteins; the sorting nexins (SNX) are also important in sorting and cargo retrieval from endosomes (Carlton *et al*., 2005; Van dergoof, 2006; Cai *et al*., 2007). Carrier peptides are small protein domains with the ability to cross biological membranes efficiently with or without specific receptors to promote the delivery of desired drug into target cells (Morris *et al*., 2001). There are examples where cell targeting ligands (CTLs) that enhance transmembrane permeation have been employed to deliver aptamers (Morris *et al*., 1997) as there is a wide variety of ligands available including antibodies, polypeptides derived from phage display libraries, and small organic molecules (Hollinger & Hudson, 2005). Cell penetrating peptides (CPPs) are small peptides of 30 amino acid residues with a net positive charge which can translocate across plasma or endomembranes, transport biomacromolecular cargos into cytoplasm and nucleus; few examples included are: R9 (arginine 9), MPG peptide, transportan, Tat, penetratin, etc. (Morris *et al*., 1997; Krissansen *et al*., 2006; Baratchi *et al*., 2010; Cheung *et al*., 2010) Several reports of noncovalent complexes between anionic siRNA chain aptamer and cationic CPPs confirm that they provided effective delivery to cells in culture (Morris *et al*., 1997; Lundberg *et al*., 2007).

### Nanoparticle systems

Aptamer technology has evolved in a multidirectional fashion finding importance and playing essential roles in fields of biosensing, imaging, drug delivery, and modern molecular medicine for therapy for various diseases and disorders. Taking the advantages of rapidly expanding nanobiotechnology-based developments; aptamer-nanoparticle conjugation forms the basis of a new chemical and biological strategy with wide application starting from assembly to detection. Because of their small size, nanoparticles can interact readily with biomolecules both on surface and within the cells and thus they are considered as a revolutionary approach for detection and therapy of various diseases. Various conjugation principles have been described leading to the application of aptamers in cancer and other inflammatory diseases as a nanodelivery system (Kanwar *et al*., 2010c). Since the aptamers are negatively charged moieties, the charge of the nanoparticle surface is very important for the conjugation between the nanoparticle and the aptamers (Farokhzad *et al*., 2004). Covalent conjugation of aptamers to nanoparticles is mainly induced by succinimidyl-ester-amine conjugations, whereas, the noncovalent conjugations include affinity interactions, metal co-ordinations, surface adsorption, etc. (Farokhzad *et al*., 2005) The conjugation can be quantitatively confirmed by various methods, such as flow cytometry, X-ray photoemission spectroscopy, Fourier transform infrared spectroscopy (FTIR), fluorescent microscopy, etc. (Hicke & Stephens, 2000). Interestingly, a chimeric phage RNA (pRNA)-aptamer (specific to CD4 antigen on T-helper cells) conjugate was synthesized and labeled with fluorescein isothyocyanate (FITC) for detection of CD4 and with this a trimeric complex of pNA-aptamer (CD4), pRNA-FITC, and pRNA-rhodamine was prepared. Using this as a nanocarrier for therapy and diagnosis (Khaled *et al*., 2005), the cellular internalization was studied with confocal microscopy. When siRNA was attached to pRNA trimer complex for silencing the CD4 surface receptor's gene expression, a decrease in CD4 expression in FITC positive cells was observed (Khaled *et al*., 2005). Another work comprised of synthesis of polylactic acid (PLA)-polyethyleneglycol (PEG) copolymers incorporated with paclitaxel (ptxl). The ptxl was incorporated into the PLA-PEG copolymer which was then attached to A10 PSMA aptamer with 5'-NH2 modifications and was tested for successful uptake in human prostate cancer cells (LNCAP and PC3) (Tong *et al*., 2010). The A10 RNA aptamer with 2'-F (fluoropyrimidine) modification was tagged to poly(lactide-co-glycolide) (PLGA) as a model-controlled release polymer system and PEG (a model hydrophilic polymer with anti-biofouling properties), to develop a triblock copolymer comprising of PLGA-PEG-A10 aptamer, which is a targeted nanodelivery system (GU *et al*., 2008).

### Nanocapsule-based targeted multidrug delivery

**Figure 4:**
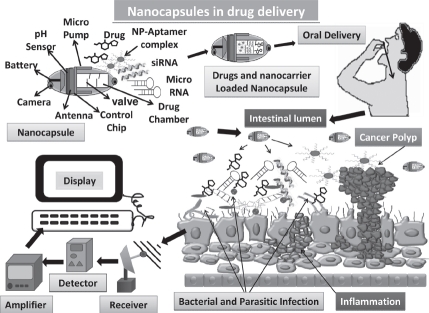
Representation of future generation drug delivery by means of nanocapsules that are equipped with real-time imaging and wireless monitoring chips. These nanocapsules can be loaded with several drugs at the same time, and can be taken orally due to lack of any toxic effects. Once present in intestine the nanocapsules can be monitored by the controlling system to release the drugs by opening the valves. The drugs can act specifically on the respective target sites due to presence of various receptors and thus multiple diseases can be targeted at the same time and can be monitored as well in real time. These drugs can be monitored by means of satellite at different places simultaneously hence providing better diagnosis by the experts (Huertas *et al*., 2010; Shen *et al*., 2010). (See colour version of this figure online at www.informahealthcare.com/bmg)

Most of these methods are well characterized and ensure appropriate detection of the desired molecule. Yet, modern developments are able to show light on the drawbacks of current methods. Nanocarriers are vehicles mainly used for drug delivery to enhance the stability and to prolong the circulation of the drug in the target tissue by promoting their permeability and retention. There are several drawbacks of nanocarriers, such as their limited drug loading capacity, difficulty in tracking the movement of the drug, imaging and diagnosis, no real-time diagnostics, lack of pH, and thermal stability. All these factors lead to lack of knowledge of mechanism of drug uptake by the cells. The mechanism of internalization of nanocarrier into target cells mainly depends on physiochemical property of the nanocarrier itself. Thermal stability and pH of nanocarrier are keys and essential factors for appropriate drug delivery. Over the time, it is evident that nanocarriers have a great potential in drug delivery. The need is to exploit, optimize, and enhance the intracellular and subcellular delivery of drugs, which are unstable or stable in physiological conditions to the target site. There is also a need to develop a multidrug nanocarrier for delivery of specific drugs for diverse functions in a particular or in various target sites. Recently multifunctional nanocapsules have emerged that are capable of providing therapy and diagnosis in real time (Figure 4). These nanocapsules are portable and thus can be used on and off the field; they possess thermal stability, pH sensibility and are capable of carrying several different drugs at the same time. They are equipped with a camera for real-time imaging. Such efficiency will help to study the intracellular fate of the various drugs as well as real-time imaging and monitoring them. These drugs can be monitored distantly as well by means of satellite by more than one personnel in different places at the same time. The release of drug can be monitored well through the computed systems and once the nanocapsules reach the target the valves can be opened in order to release the drugs. Use of biodegradable nanocapsules will minimize the chance of drug toxicity and size of nanocapsules can be regulated considering the amount of drug to be loaded. The aptamer will bind to the target due to its high specificity, whereas the other drugs will bind to their respective target due to expression of various markers and receptors. This development will increase the possibility of targeting the nanocarriers to the desired cells and to study the nanocarrier cell interaction (Hillaireau & Couvreur, 2009; Huertas *et al*., 2010; Shen *et al*., 2010; Wang & Ho, 2010b).

### Multitargeted LNA-aptamer complexes with anticancerloaded iron-saturated bovine lactofeerin dopamine surface modified Fe_3_O_4_ nanoparticles for specific killing of tumor cells

**Figure 5:**
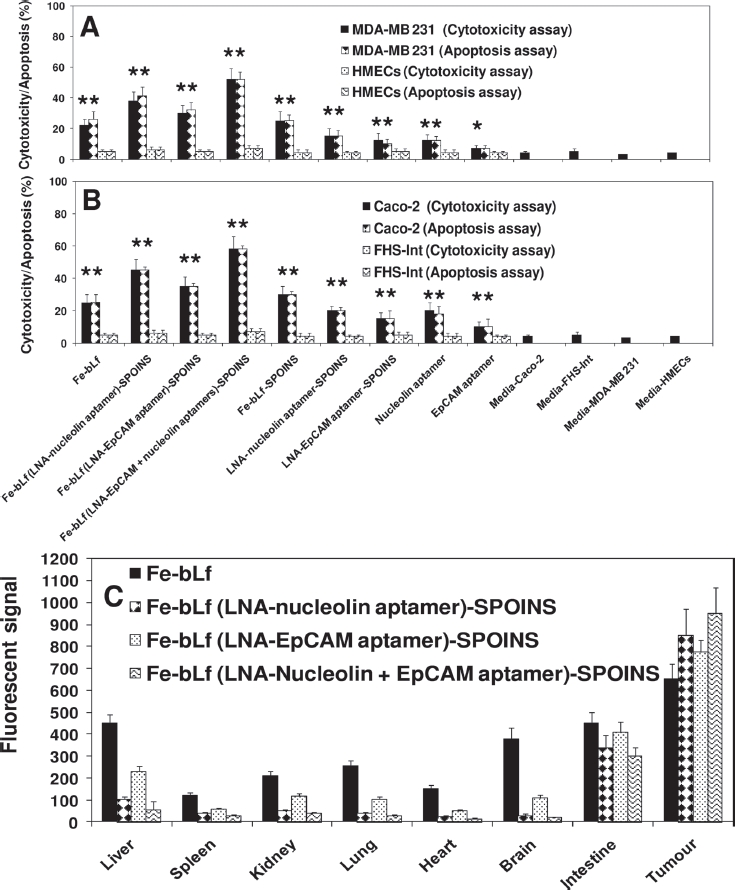
Cell cytotoxicity determined by LDH release assay and cell death (apoptosis) by TUNEL assay of (A) breast cancer cells and (B) colon cancer cells and compared with normal cells following treatment with 1600 jUg/mL of iron saturated lactoferrin (Fe-bLf) and other control nanoformulations and control aptamers (nucleolin and EpCAM). Cells were treated for 24 h with different nanoformulations and stained by TUNEL analysis for apoptotic cells. Cell death is shown here in terms of apoptotic (% apoptosis) and LDH release (% cytotoxicity). All treatments were performed in triplicate and assay was repeated three times independently with similar results. The mean for representative experiment was calculated and presented as a mean ± SD values; ** indicates a highly significant p< 0.001 value from the normal control cell lines and with media only; * indicates a significant p< 0.05 value from the normal control cell lines and control with media only, (C) Biodistribution study of nanocarriers compared with Fe-bLf in oral administrations. Fluorescent signal of tissue extracts after 36 h of oral administration. Nanocarriers were labeled with coumarin (10mg/mL).

We showed that 100% iron-saturated bovine lactofeerin (Fe-bLf) acts as a potent natural adjuvant and fortifying agent for augmenting cancer chemotherapy with broad utility in the treatment of cancer (Kanwar *et al*., 2008). The key findings of the study revealed: (i) Fe-bLf bound to the intestinal epithelium and was preferentially taken up within Peyer's patches(Figure 2); (ii) Fe-bLf enhanced antitumor activity in combination with major anticancer drugs (paclitaxel, doxorubicin, epirubucin, or flurouracil), the combination being capable of completely eradicating large tumors of EL4 lymphoma, Lewis lung carcinoma, B16 melanoma (0.6cm diameter) that were otherwise completely insensitive to chemotherapy; (iii) Natural bLf, 4% iron-saturated or 50% iron-saturated bLf were less effective at potentiating cancer chemotherapy, and did not cause tumor eradication; (iv) Fe-bLf increased the production of Th1 and Th2 cytokines within the intestine and tumor, including TNF-α, IFN-γ, as well as nitric oxide (NO) that have been reported to sensitize tumors to chemotherapy; (v) Fe-bLf almost completely reduced tumor vascularity (angiogenesis) and blood flow, and increased tumor apoptosis regulated by survivin, Bcl-2, caspases 9/3, and Fas molecules; (vi) Fe-bLf increased leukocyte infiltration (CD4^+^,CD8^+^, NK, and dendritic cells) to tumors, lamina propria, and spleen; and (vii) importantly, Fe-bLf restored both red (RBC) and white (WBC) blood cell numbers depleted by chemotherapy, potentially fortifying the mice against cancer (Kanwar *et al*., 2008; Gibbons *et al*., 2011; Kanwar RK *et al*., 2011). The Fe-bLf was prepared (Kanwar *et al*., 2008). Superparamagnetic iron oxide (Fe_3_O_4_) nanoparticles (SPIONs), which are nontoxic, biodegradable, inexpensive and candidate platforms for the build-up of theranostic nanostructures were prepared as described (Xie *et al*., 2010, Soundararajan *et al*., 2008). Here, we used dopamine to modify the surface of SPIONs for yielding nanoconjugates that can be easily encapsulated into anticancer Fe-bLf protein. These nanocarrier systems were well-suited for dual encapsulation of SPIONs for imaging or monitoring therapy and drug molecules, because the encapsulation or conjugation is achieved in a way that is similar to common drug loading. The LNA-nucleolin DNA aptamer (molecule present on cancer cell surfaces and translocated to nucleus) (Soundararajan *et al*., 2008) and LNA-EpCAM RNA aptamer (Shigdar *et al*., 2011), known to be over-expressed on the apical side of the cancer cell surfaces were prepared (Hertoghs *et al*., 2003) and loaded individually or in combination on these Fe-bLf-SPOINS nanocarriers. The average size of the nanocarriers in various steps of their preparations were found to be (170±35 nm) as determined by dynamic light scattering (DLS), scanning electron microscopy (SEM), and transmission electron microscopy (TEM), Fourier transform infrared spectroscopy (FTIR), differential scanning calorimeter (DSC), thermogravimetric analysis (TGA), and X-ray diffraction (XRD). These LNA-aptamers-loaded Fe-bLf nanocarriers were treated with human cancer cell line Caco-2 (human colon cancer), MDA-MB 231 (human breast cancer), normal HMECs (human mammary epithelial cells), and normal FHs 74 Int (an adherent human primary fetal small intestinal) cell lines obtained from the American Type Culture Collection (ATCC, USA). These LNA-aptamer complexes of Fe-bLf nanocarriers induce massive cell death in breast and colon cancer cells *in vitro* and spare normal HMECs and FHs 74 Int cells in an *in vitro* co-culture model (Figure 5A and B). A rotational magnetic field frequency of 1Hz has shown a maximum amount of LDH release or cytotoxicity and cell death through apoptosis measured by Tunnel and Annexin-V positive cells compared to normal cells (Kanwar *et al*., 2001; Kanwar *et al*., 2008). After applying magnetic field with 1Hz, we found that the cancer cells massively increased in breast and colon as compared to normal HMECs and normal FHs 74 Int cell lines (data not shown). This indicates that SPIONs-loaded LNA-aptamers have the potential to kill cancer cells more specifically, effectively, and spare normal cells. We evaluated the tissue biodistribution of these nanocarriers using a fluorescent marker, 6-coumarin and the fluorescent quantum yield characters enabled the selective tracking of the *in vivo* distribution of nanocapsule (Woodrow, 2009) (Figure 5C). These signals were captured by magnetic sensors and release of the Fe-bLf -loaded LNA-aptamers from nanocarriers was monitored on computer from outside (unpublished information). The release of nanocarriers can be controlled in *in vivo* situations not only in human gut associated microbial (parasitic or viral or bacterial) infections, inflammations, and cancers but also anywhere in the body by LNA-aptamers targeting nanocarriers (Figure 4). Thus, we developed LNA-modified nucleolin aptamers and LNA-modified EpCAM aptamers conjugated “Fe-bLf natural anticancer protein-loaded nanobullet nanocarriers,” which specifically target cancer cells and spare normal cells. Hence, we developed natural nanomedicinal-based war against cancer cells with targeted nanobullet nanocarriers that specifically induce their traumatic death and spare normal cells. Superparamagnetic iron oxide (Fe_3_O_4_) nanoparticles (SPIONs) nature of these nanocarriers was used to monitor the size of tumors in the treated mice through different imaging systems such as *in vivo* positron emission tomography (PET)/near-infrared fluorescence (NIRF)/magnetic resonance imaging (MRI) for future potential contrast material for cancer diagnosis as described earlier (Xie *et al*., 2010).

### Aptamers in clinical trials

With new developments in nanotechnology and chemistry, few aptamers have found their way into modern medicine. The pharmaceutical industries are ready to invest more on recent nano-aptamer technology which has added benefits when compared to the traditional drugs. In a very short span of time, aptamers have attained critical importance in the field of modern molecular medicine and nanomedicine. Several aptamers are in clinical trial such as, EYE001 targeting vascular endothelial growth factor receptor (VEGFR); phase II/III (Carrasquillo *et al*., 2003). Edifoligide aptamer which is used to treat vein graft failure of heart and leg; phase III (C. Inc, 2004). The E100300 to target platelet derived growth factor (PDGF) (phase II) (JO *et al*., 2006). The Nu172 to target thrombin; phase II (Wagner-Whyte *et al*., 2007), REG1, which targets factor IXa; phase IIb (Becker, 2009), ARC 1779, which inhibits the acute coronary syndromes by preventing the binding of platelet receptor glycoprotein Ib to its receptor; phase I/II (ClinicalTrials.gov, 2009), AS1411, which is being used to treat cancer by inhibiting their DNA replication; phase III (Stuart *et al*., 2009), ARC1905, which prevents age-related macular degeneration (AMD); phase I (ClinicalTrials.gov, 2010), LY2181308, which is used in treatment of nonsmall cell lung cancer (NSLC); phase III (Cancerhelp.org.uk, 2010), and many others. However, macugen was the first aptamer to get the FDA approval in 2004 for treatment of wet AMD (NG *et al*., 2006).

## Conclusion

Various chimeras such as aptamer-aptamer, aptamer-siRNA, aptamer-miRNA, aptamer-enzyme, aptamer-antibody, and aptamer-NRTI have been established. All of these chimeras show better stability and functional capability than the parent molecules used to form the chimera. Several methodologies have been established to produce and select the chimeras based on their specificity such as chimeric SELEX. Specific delivery of chimeric aptamers has been achieved by their conjugation with various nanoparticles and according to the nature of nanoparticles or the modifications in the aptamer chimeras and can be used for imaging and diagnosis. Aptamer chimerism offers a wide range of activity due to the presence of two molecules playing two or more different roles simultaneously. They have an additional advantage over the existing methodology and are highly stable, more flexible with various types of modifications, and cost effective. Since aptamers are nonimmunogenic, most of the aptamer chimerics can be expected to be less or nonimmunogenic. Aptamer chimerics have various applications, such as tumoricidal activity, detection of small analytes, HIV inhibitory properties and detection of pathogens in food samples, personalized drug and vaccine development against parasites, bacterial, and viral agents, targeting cancer cells at a subcellular level, such as the cell organelles, and targeting different cellular cascade pathways. Well-characterized chimerics can be brought into therapeutic use for various inflammatory disorders as well. Because aptamer chimeras can be generated for several biomarkers and receptors, they offer a better specificity, target recognition, and improved therapeutics than any antibodies or synthetic peptides. Recently, various drug delivery and imaging systems have been developed which can be further improved with the help of aptamer chimerics. Nanocapsules with modern technologies for real-time imaging can be used to monitor the interaction between the aptamer chimeras and target cells. Multivalent nanoparticles, such as dendrimers with high potential for performing several functions simultaneously can be developed by the use of chimeric aptamers. Thus, aptamer chimeras may prove to be useful in future for a wide range of diseases.

## Declaration of interest

The work was supported by the grants from Institute of Biotechnology, Institute for Technology and Research Innovation and the Australia-India Strategic Research Fund (AISRF BF030016).
